# Approval of Cancer Drugs With Uncertain Therapeutic Value: A Comparison of Regulatory Decisions in Europe and the United States

**DOI:** 10.1111/1468-0009.12476

**Published:** 2020-10-06

**Authors:** MAXIMILIAN SALCHER‐KONRAD, HUSEYIN NACI, COURTNEY DAVIS

**Affiliations:** ^1^ London School of Economics and Political Science; ^2^ King's College London

**Keywords:** pharmaceutical regulation, US Food and Drug Administration, European Medicines Agency, cancer

## Abstract

**Policy Points**

- Regulatory agencies may have limited evidence on the clinical benefits and harms of new drugs when deciding whether new therapeutic agents are allowed to enter the market and under which conditions, including whether approval is granted under special regulatory pathways and obligations to address knowledge gaps through postmarketing studies are imposed.
- In a matched comparison of marketing applications for cancer drugs of uncertain therapeutic value reviewed by both the US Food and Drug Administration (FDA) and the European Medicines Agency (EMA), we found frequent discordance between the two agencies on regulatory outcomes and the use of special regulatory pathways. Both agencies often granted regular approval, even when the other agency judged there to be substantial uncertainty about drug benefits and risks that needed to be resolved through additional studies in the postmarketing period.
- Postmarketing studies imposed by regulators under special approval pathways to address remaining questions of efficacy and safety may not be suited to deliver timely, confirmatory evidence due to shortcomings in study design and delays, raising questions over the suitability of the FDA's Accelerated Approval and the EMA's Conditional Marketing Authorization as tools for allowing early market access for cancer drugs while maintaining rigorous regulatory standards.

**Context:**

Regulatory agencies are increasingly required to make market approval decisions for new drugs on the basis of limited clinical evidence, a situation commonly encountered in cancer. We aimed to investigate how regulators manage uncertainty in the benefit‐risk profiles of new cancer drugs by comparing decisions for the world's two largest regulatory bodies—the US Food and Drug Administration (FDA) and the European Medicines Agency (EMA)—over a 5‐year period.

**Methods:**

We systematically identified a set of cancer drug‐indication pairs for which data on efficacy and safety was less complete than that required for regular approval at time of market entry from 2009 to 2013, as determined by the FDA's use of Accelerated Approval (AA) or the EMA's use of Conditional Marketing Authorization (CMA) pathways, and matched these across the two agencies. Using publicly available information, we compared regulatory pathways and outcomes, final approved indications, and postmarketing obligations imposed by the agencies.

**Findings:**

We identified 21 cancer drug‐indication pairs that received FDA AA, EMA CMA, or both. Although most applications relied on identical pivotal trials across the FDA and the EMA, regulatory pathways often differed; 57% of indications received either FDA AA or EMA CMA, and regular approval by the other agency. After approval, the EMA more often accepted single‐arm studies to confirm clinical benefit compared to the FDA (75% vs. 29% of indications), and the FDA more commonly requested randomized controlled trials (85% vs. 50%). Forty‐one percent of confirmatory trials after FDA AA were conducted in different populations than the approved indication, compared to 13% after EMA CMA. Both agencies relied primarily on surrogate measures of patient benefit for postmarketing obligations. After a median follow‐up of 7.25 years, 40% of FDA and 61% of EMA postmarketing obligations after AA and CMA, respectively, were delayed.

**Conclusions:**

US and European regulators often deemed early and less complete evidence on benefit‐risk profiles of cancer drugs sufficient to grant regular approval, raising questions over regulatory standards for the approval of new medicines. Even when imposing confirmatory studies in the postmarketing period through special approval pathways, meaningful evidence may not materialize due to shortcomings in study design and delays in conducting required studies with due diligence.

In the united states (us) and the european union (eu), regulatory agencies—the US Food and Drug Administration (FDA) and the European Medicines Agency (EMA), respectively—are responsible for assessing the benefit‐risk profile of new drugs before they enter the market. The mandate of these regulators to protect public health by ensuring that patients have access to safe and efficacious drugs requires them to carefully balance the need for robust and comprehensive evidence on efficacy and safety at the time of market approval while making promising new drugs available to patients in a timely manner. This “evidence vs. access conundrum”^1^ can also be framed in terms of the amount of uncertainty regulators are willing to accept. Placing a greater weight on the availability of complete evidence for regulatory decision making, typically obtained from randomized controlled trials (RCTs) that measure patient‐relevant endpoints such as survival, means more certainty that drugs available on the market have a positive benefit‐risk profile. Conversely, granting approval on the basis of incomplete evidence shows greater willingness to accept uncertainty about a new drug's therapeutic value.

Beginning in the 1980s—in response to pressure from the pharmaceutical industry and some patient advocates, and as instructed by the US Congress—the FDA introduced special regulatory provisions that aimed to shorten the time to market approval for products intended to treat patients with serious illnesses.^2^ These provisions either expedite the regulatory *review process* by reducing the time available for regulators to review new applications (Priority Review, introduced in 1992), or they expedite the premarket drug *development process* by allowing pharmaceutical manufacturers to submit preliminary (or immature) evidence on efficacy and safety (Accelerated Approval, or AA, introduced in 1992).^3^

The EMA, following the example of the FDA, introduced similar provisions that either shortened regulatory review times (Accelerated Assessment, introduced in 2004; PRIME, 2016) or changed evidentiary standards compared to regular approval (Approval Under Exceptional Circumstances, 1995; Conditional Marketing Authorization, or CMA, 2006).^4^ In both the United States and the EU, these special regulatory programs most commonly benefit cancer drugs.^5^, ^6^, ^7^ While they appear to have succeeded in reducing the time until marketing authorization in the United States^8^—although not in Europe^9^—a body of literature is emerging that questions their benefits to patients. Research on special approval pathways has pointed toward questionable novelty of drugs that benefited from these programs,^6^, ^10^ a higher number of safety events in the United States (although not in Europe),^11^, ^12^ and an erosion of the evidence landscape, with robust evidence on the efficacy and safety of new medicines often unavailable at the time of marketing authorization and unlikely to become available in a timely manner in the postapproval phase.^13^, ^14^, ^15^, ^16^

Whilst the programs discussed here all aim to reduce the amount of time it takes for drugs to be approved onto the market, AA in the United States and CMA in the EU are particularly illustrative from a regulatory perspective because they shift part of the evidence generation for regulatory decision making from the preapproval to the postapproval period. This allows drugs to enter the market based on evidence that regulators consider to be preliminary and in need of further substantiation. First introduced in the United States in 1992, AA allows early approval of drugs that address an unmet medical need in the treatment of a serious condition and provide a meaningful advantage over available therapeutic options. AA is granted on the basis of a surrogate measure that is considered to be “reasonably likely” to predict clinical benefit but has not been validated, and includes an obligation to generate confirmatory evidence of this benefit through postmarketing studies.^17^ Although the preliminary and limited nature of the evidence submitted at the time of initial approval introduces uncertainty, this is, in theory, addressed through mandatory postmarketing studies. In practice, research has shown that uncertainty may persist for years after initial approval due to design limitations of postmarketing studies (including continuing reliance on surrogate measures^18^) and/or their untimely completion.^14^

In the EU, the CMA pathway largely follows the same principle as AA, as it allows the EMA to grant approval conditional on complying with obligations to generate additional evidence in the postmarketing phase.^19^ In both markets, decisions to pursue approval via the AA or CMA pathway are often made late in the process of bringing a drug to market,^9^, ^20^ and, in the case of the EMA, are seen by the regulator as a “rescue option” to grant approval when available evidence is insufficient for regular approval. Important differences between EMA's CMA and FDA's AA include that only first marketing authorizations of new products are eligible for CMA, whereas the AA pathway is also available for additional indications of approved medicines, and that CMA is reviewed annually until all obligations are fulfilled. The vast majority of drugs approved through CMA and AA pathways are cancer drugs.^7^, ^14^

Although formal criteria for drug approval are specified in law and regulation (see Box for an overview of AA and CMA provisions), in practice regulators, in negotiation with companies, are afforded some flexibility when determining what constitutes an acceptable level of uncertainty^21^ and may use a range of regulatory tools to manage situations where evidence on efficacy and safety is limited. If regulators judge the magnitude of uncertainty to be such that it precludes regular approval, they may grant approval under a special pathway (AA in the United States or CMA in the EU) but impose postmarketing study obligations to address critical knowledge gaps as described earlier. Regulators may also restrict the approved indication to a specific subset of the studied population for which more robust evidence exists. Alternatively, regulators may decide there is insufficient evidence to support either regular, special, or some form of restricted approval, and refuse marketing authorization.

Box. Comparison of Special Approval Pathways in Europe (Conditional Marketing Authorization, CMA) and the United States (Accelerated Approval, AA)

**Table:** 

	EMA Conditional Marketing Authorization^19^	FDA Accelerated Approval^17^
Key features	Requires four criteria to be fulfilled, including a positive benefit‐risk profile and the ability to provide comprehensive data through postmarketing studies Marketing authorization is reviewed every year until conditions of initial authorization are fulfilled Can only be granted for first marketing authorizations	Allows market entry of drugs on the basis of a surrogate endpoint that is “reasonably likely” to predict clinical benefit (as opposed to a validated surrogate endpoint) Clinical benefit is to be confirmed through postmarketing trials Can be granted for first marketing authorizations as well as further indications for approved medicines
Eligibility criteria	Medicinal products that fulfill one of the following: Treat, prevent, or diagnose seriously debilitating or life‐threatening diseases Are used in response to public health emergency situations Are designated as orphan medicinal products	Drugs and biologics that fulfill all of the following: Address an unmet medical need in the treatment of a serious condition Provide a meaningful advantage over available therapies (where a therapy exists) Have been shown to be effective on the basis of an endpoint “reasonably likely” to predict clinical benefit
Evidence assessment	Requires that four criteria are fulfilled: Positive benefit‐risk ratio is established The manufacturer is likely to be able to provide additional data The product fulfills an unmet medical need Benefits to public health of immediate availability of the product outweigh the risks of incomplete evidence	The same statutory requirements as for regular approval must be met: Substantial evidence on efficacy based on adequate and well‐controlled trials Sufficient information to determine the drug is safe However, the evidence standard is different: Efficacy can be demonstrated on the basis of a surrogate endpoint that was determined to be “reasonably likely” to predict clinical benefit FDA accepts that evidence will generally come from fewer, smaller, and shorter trials
Theoretical consequences for failure to comply with conditions	Failure to comply with postmarketing obligations will lead to an assessment report by the EMA, which can result in one of the following:^22^ Letter to the marketing authorization holder (i.e., the pharmaceutical company) Oral explanation by the marketing authorization holder Initiation of procedure to vary, suspend, or revoke approval Inspection	Failure to conduct postmarketing studies “with due diligence” may result in withdrawal of approval Violation of postmarketing obligations may also result in civil monetary penalties of up to US $250,000^23^

How these different regulatory tools are used by the FDA and the EMA to manage situations with substantial uncertainty about the benefits and harms of a new drug is not adequately researched. The majority of previous studies of cancer drug approvals had a primary focus on one of the two agencies.^18^, ^20^, ^24^, ^25^, ^26^ Although some studies documented regulatory decision making under uncertainty, they were limited to the perspective of a single regulatory agency.^9^, ^21^ Comparative studies of the FDA and the EMA mainly examined differences in time to market approval^9^, ^27^, ^28^, ^29^, ^30^ or focused on the use of individual regulatory tools such as approval decisions or wording of approved indications.^29^, ^31^, ^32^ Previous studies have not systematically compared the use of a comprehensive set of regulatory tools by the FDA and the EMA in situations with limited clinical evidence about the benefit‐risk profile of new drugs. Therefore, important gaps remain to characterize the decisions made by regulators under such circumstances, including whether approval is granted, which approval pathway is used, whether wording of final approved indications have restrictions, and which additional evidence regulators require to be generated.

We aimed to analyze how the FDA and the EMA deal with uncertainty by comparing regulatory outcomes (approval vs. no approval decision), pathways (regular approval vs. approval through a special pathway), final approved indications, and regulator‐imposed obligations to generate additional evidence in the postmarketing phase, for a matched set of cancer drug‐indication pairs with less complete data at time of market entry than usually required for regulatory approval. We focus on cancer drugs, since this is the therapeutic area with the largest proportion of drug approvals benefiting from special regulatory pathways and with the highest discrepancy in regulatory approval decisions between these two regulatory bodies.^31^

## Methods

We compared the EMA's and the FDA's handling of uncertainty in four steps. First, we systematically identified a set of cancer drug‐indication pairs that were judged by regulators to have limited evidence on efficacy and safety at the time of approval and still required confirmation. We used approval through either the EMA's CMA or the FDA's AA pathway to indicate drugs for which at least one of the two regulators considered the uncertainty about efficacy and safety at the time of market entry to be substantial enough to preclude regular approval. Second, we matched this set of drug‐indication pairs across the EMA and the FDA. Third, we reviewed publicly available regulatory documents to compile information from the EMA and the FDA on regulatory pathways and outcomes, final approved indications, pivotal trial characteristics, and postmarketing obligations for each drug‐indication pair. Fourth, we compared the compiled information between the EMA and the FDA. We describe each step in more detail in this section.

From all cancer drug indications approved by the EMA (from inception of the CMA pathway in 2006 until 2016) or the FDA (from inception of the AA pathway in 1992 until 2017), we identified those that were approved through either the EMA CMA or the FDA AA pathway from published reports.^7^, ^18^ This list included all cancer drugs for which at least one of the two agencies considered the evidence at the time of approval less complete than required for regular approval. For each of the drug‐indication pairs on this list, we then conducted targeted database searches on the EMA and FDA websites to identify the closest matching drug‐indication pairs with a regulatory outcome by the other agency. At a minimum, indications had to match on cancer type (e.g., lung cancer, breast cancer, haematologic malignancies), followed by line of treatment, single agent and combination therapy, and population (e.g., type of tumor, biomarkers, histology), if possible. For example, if a drug‐indication pair was included in the list because it received EMA CMA, we searched all FDA regulatory outcomes for the name of the drug and then compared whether EMA and FDA indications matched on cancer type, followed by other characteristics. If no better match based on the parameters could be identified, we considered EMA‐FDA drug‐indication pairs as matches as long as the cancer type was the same. Thus, a match could be considered for the same drug indicated for first‐line treatment by one agency and second‐line treatment by the other agency, as long as no regulatory decision about the same‐line treatment was available from both agencies. Differences in final approved indications between matched drug‐indication pairs were documented.^29^

For the EMA, database searches allowed us to identify drugs that were approved, drugs that were denied approval, and drug marketing authorization applications withdrawn by the manufacturer in anticipation of a negative outcome before a final recommendation was formed. For the FDA, database searches allowed us to systematically identify only those drugs that were approved. When a drug was not found in the FDA database, we conducted additional web searches and made use of publicly available documents (such as committee meeting minutes) for information about whether an application had been submitted.

We restricted this set of drug‐indication pairs to those with an EMA regulatory outcome (approval, no approval, or application withdrawn by the manufacturer) between 2009 and 2013. The time period was chosen to allow for sufficient time for postmarketing obligations to be completed (minimum of 5 years postapproval until we conducted data collection in December 2018). We excluded regulatory outcomes for generics, treatments of benign tumors, supportive treatments, and advanced medicine therapy products such as gene or cell therapies.

For each matched drug‐indication pair, we compiled information on the regulatory outcome and pathway, current status, final approved indication, pivotal trial(s) at the time of initial approval, and postmarketing obligations and their current status from various sources. Information relating to the regulatory assessment procedure was extracted from publicly available documents at the EMA (European Assessment Reports, or EPARs, accessed through the EMA website) and the FDA (review documents, label, approval letter, and administrative and correspondence documents, accessed through the Drugs@FDA database) and included information on regulatory outcome and pathway, submission and decision dates, orphan drug designation status, and whether the application was for a first marketing authorization or a supplemental approval (variation) of a previously approved drug. Pivotal trial characteristics for most drug‐indication pairs were available from two previous studies of all FDA and EMA accelerated approvals.^14^, ^24^ For drug‐indication pairs not included in these studies, we extracted relevant information on EMA and FDA pivotal trials from EPARs and review documents and labels, respectively. Where an application for marketing authorization included more than one indication for the same drug, we treated these as separate drug‐indication pairs in order to assess the evidence submitted and collected in the postmarketing setting for each indication.

We extracted information on postmarketing studies that were required by the regulator to confirm the product's efficacy and safety, which we collectively refer to as postmarketing obligations. These included specific obligations under CMA as well as Annex II conditions for EMA approvals; and AA Subparts E and H, as well as section 505(o) requirements, for FDA approvals. We did not extract information on studies that formed part of routine pharmacovigilance activities, that were conducted voluntarily by the manufacturer, or that were conducted in animals or cells. Further, we categorized studies as either focusing on confirmation of clinical efficacy and safety or investigating other issues (e.g., pharmacokinetics and dosing studies), and we report results only for the former. We further assessed whether clinical efficacy and safety studies were conducted in a patient population similar to the approved indication. We categorized postmarketing study populations as similar to the approved indication when the study was conducted in patients who could receive the drug according to its approved label or when the study was a follow‐up of the pivotal trial. Postmarketing study populations were categorized as dissimilar to the approved indication when there were obvious differences, such as a different cancer type, and when the line of treatment was different (e.g., postmarketing study is conducted in previously untreated patients when the approved indication is for second‐line treatment). A postmarketing obligation was considered fulfilled when the regulator removed it from the list of required studies, including cases in which the manufacturer was released from the obligation without submitting the results from a completed study. Additional information on the compilation of information on postmarketing obligations and their status is provided in the Online Appendix.

In the next section, we present key features of the regulatory decisions—including regulatory outcome, pathway, pivotal trial evidence, final approved indication, and postmarketing obligations—for each matched drug‐indication pair and describe differences and similarities between the EMA and the FDA.

## Results

**Figure 1:**
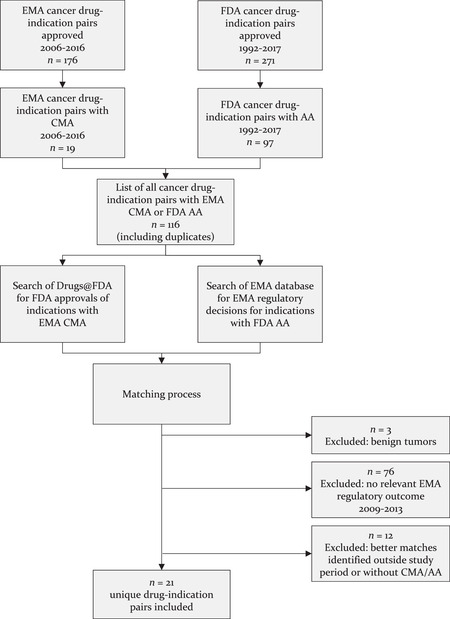
Flow Chart of Selection Process for Matched Drug‐Indication Pairs

We identified a total of 116 cancer drug‐indication pairs with limited clinical data on efficacy and safety, as indicated by approval granted through either EMA CMA (*n* = 19; 11% of all approved EMA cancer drug indications from 2006 to 2016) or FDA AA (*n =* 97; 36% of all approved FDA cancer drug indications from 1992 to 2017) pathways. After matching these with all other available EMA and FDA decisions and excluding matched pairs without an EMA regulatory outcome between 2009 and 2013, we arrived at a sample of 21 unique matched drug‐indication pairs of cancer products that received approval through the EMA's CMA or the FDA's AA pathway (Figure 1).

Table 1 provides an overview of regulatory pathways and outcomes of the 21 matched drug‐indication pairs. Compared to the FDA, the EMA more often granted regular approval (seven [33%] vs. five [24%] of matched drug‐indication pairs, respectively) or did not grant approval (four [19%] vs. one [5%]), whereas the FDA more often utilized AA (15 [71%]) compared to the EMA using CMA (10 [48%]). Overall, there was little overlap in the use of special regulatory pathways at the EMA and the FDA. Only four (19%) matched drug‐indication pairs received approval through both the EMA CMA and the FDA AA pathways. The most common combination of regulatory pathways was regular EMA approval and FDA AA (seven indications [33%]), followed by the reverse, EMA CMA and regular FDA approval (five indications [24%]). A further four (19%) indications were not approved by the EMA but benefited from FDA AA, and one indication with EMA CMA approval was not approved by the FDA.

**Table 1:** Combinations of Regulatory Pathways and Outcomes in 21 Matched Cancer Drug‐Indication Pairs (Number of Drug‐Indication Pairs, Percent of Total Sample)

	**FDA Regular Approval**	**FDA AA**	**No FDA Approval**	**Total**
**EMA Regular Approval**	—	7 (33%)	—	**7 (33%)**
**EMA CMA**	5 (24%)	4 (19%)	1 (5%)	**10 (48%)**
**No EMA Approval**	—	4 (19%)	—	**4 (19%)**
**Total**	**5 (24%)**	**15 (71%)**	**1 (5%)**	**21 (100%)**

Abbreviations: AA, Accelerated Approval; CMA, Conditional Marketing Authorization; EMA, European Medicines Agency; FDA, US Food and Drug Administration.

Sixteen of 21 matched drug‐indication pairs were first marketing authorizations (Table 2). The remaining five indications were variations of prior approvals and therefore not eligible for EMA CMA. Four of the five variations were approved under the regular EMA and FDA AA pathways. As of December 2018, half of the indications with EMA CMA had been converted to full (regular) approvals. The other half (*n =* 5) remained under CMA provisions. Conversely, for 13 of 15 indications with FDA AA, all postmarketing obligations had been fulfilled by December 2018.

**Table 2:** Characteristics of Matched Drug‐Indication Pairs

	**EMA**	**FDA**
	**Number of indications (%)**	
**Matched Drug‐Indication Pairs, Total**	**21**	**21**
Orphan designation	14 (67%)	15 (71%)
First marketing authorization	16 (76%)	16 (76%)
*Current status (December 2018)*		
Fully approved	12 (57%)	18 (86%)
Remains under special approval (CMA/AA)	5 (24%)	2 (10%)
Not approved	4 (19%)	1 (5%)

Abbreviations: AA, Accelerated Approval; CMA, Conditional Marketing Authorization; EMA, European Medicines Agency; FDA, US Food and Drug Administration.

### Comparison of Regulatory Pathways and Outcomes with Pivotal Trial Evidence

To further investigate the discrepancies in the use of regulatory pathways between the two agencies and explore possible explanatory factors, such as availability of more robust efficacy and safety data, as well as statutory restrictions on the use of EMA CMA, we compared the approval pathways, pivotal trial evidence, and final approved indications for each of the 21 matched drug‐indication pairs (Table 3).

**Table 3:** Comparison of Approval, Regulatory Pathway, Pivotal Trial Design, and Approved Indication for Matched Drug‐Indication Pairs

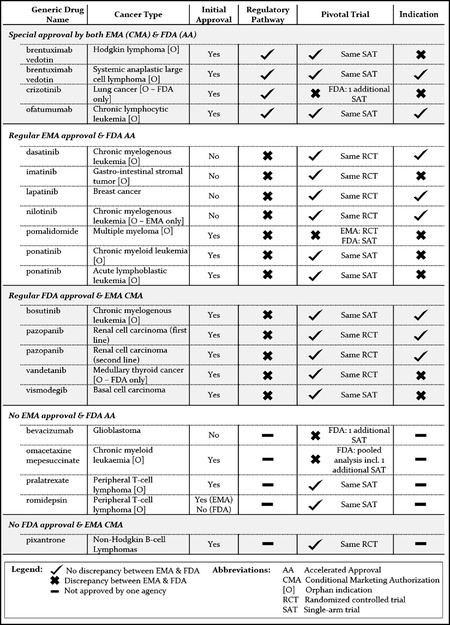

Overall, regulatory decisions for 17 of the 21 (81%) matched drug‐indication pairs were based on the same pivotal trials. For the remaining four drug‐indication pairs, differences in pivotal trials were due to the EMA considering a trial included in the evidence package as “supportive” that was labeled “pivotal” by the FDA in two cases, and due to different trials being submitted to the EMA and the FDA in the other two cases.

Regular EMA approvals with matching FDA AA were more often based on RCTs (five of six pivotal trials [83%]) compared to regular FDA approvals with matching EMA CMA (two of four pivotal trials [50%]).

None of the pivotal trials submitted for 21 marketing authorization applications at the two agencies used overall survival as a primary endpoint. Indications approved through the EMA's CMA and the FDA's AA pathways were predominantly based on trials with response rate endpoints, although EMA CMAs also relied on progression‐free survival (PFS) or recurrence‐free survival in three of 10 trials (30%) compared to two of 17 trials (12%) in FDA approvals with AA. Both the EMA (in four cases) and the FDA (in two cases) granted regular approval on the basis of trials with response endpoints for matched drug‐indication pairs that were approved through special pathways (CMA or AA) by the other agency.

Among indications approved through both the EMA CMA and FDA AA pathways (*n =* 4), there were no meaningful differences in the final approved indications in two cases, and the EMA used a more restrictive final approved indication for the remaining two.

Among indications with regular EMA approval and FDA AA (*n =* 7), the EMA used more restrictive wording in three cases that were based on the same pivotal trial evidence. For pomalidomide, the EMA's regular approval was restricted to combination therapy with dexamethasone, mirroring how the drug was studied in the pivotal trial accepted by the EMA. Four of the seven drug‐indication pairs with regular EMA approval and FDA AA were variations of existing approvals (dasatinib, imatinib, laptinib, nilotinib), therefore excluding the possibility of EMA CMA, and three (pomalidomide and two ponatinib indications) were initial marketing authorization applications, without statutory restrictions regarding the regulatory pathway. The EMA used more restrictive wording for the three initial marketing authorizations, but for only one of the four variations.

### Comparison of Postmarketing Obligations

**Figure 2:**
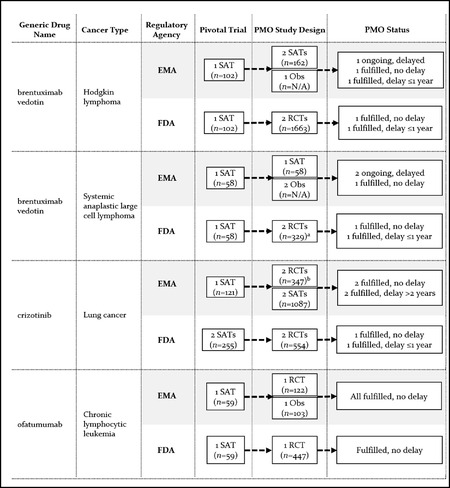
Premarketing and Postmarketing Evidence Requirements and Status of Clinical Postmarketing Obligations (PMOs) for Matched Drug‐Indication Pairs With EMA CMA and FDA AA Abbreviations: N/A, sample size not available because study is ongoing; Obs, observational phase IV study; PMO, postmarketing obligation; RCT, randomized controlled trial; SAT, single‐arm trial. ^a^ Sample size is for one RCT only, as the obligation for the other RCT was considered fulfilled through a related obligation in the Hodgkin lymphoma indication. ^b^ Two obligations relating to the same RCT.

**Figure 3:**
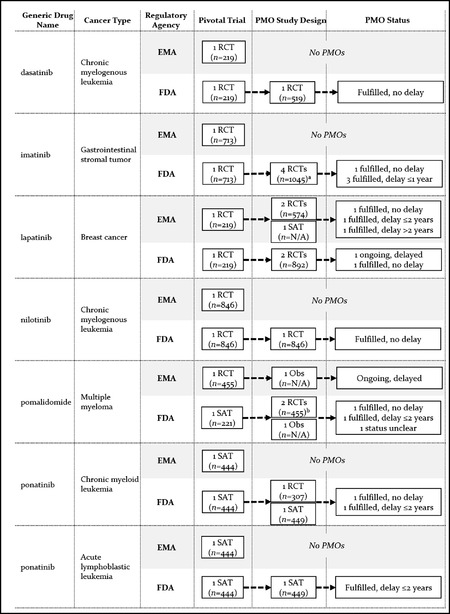
Premarketing and Postmarketing Evidence Requirements and Status of Clinical Postmarketing Obligations (PMOs) for Matched Drug‐Indication Pairs With Regular EMA Approval and FDA AA Abbreviations: AA, FDA Accelerated Approval; N/A, sample size not available because study is ongoing; Obs, observational phase IV study; PMO, postmarketing obligation; RCT, randomized controlled trial; SAT, single‐arm trial. ^a^ Three obligations relating to the same RCT. ^b^ Sample size relates to one RCT only. Obligation for the other RCT was considered fulfilled through another study.

**Figure 4:**
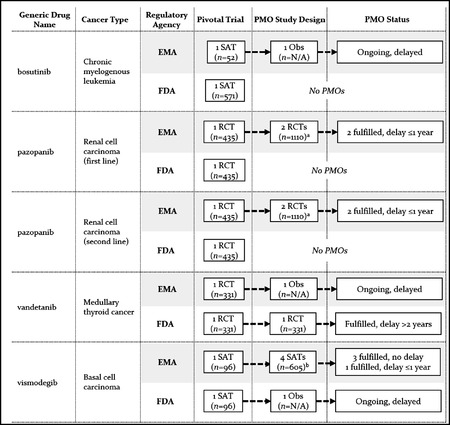
Premarketing and Postmarketing Evidence Requirements and Status of Clinical Postmarketing Obligations (PMOs) for Matched Drug‐Indication Pairs With Regular FDA Approval and EMA CMA Abbreviations: CMA, European Medicines Agency Conditional Marketing Authorization; N/A, sample size not available because study is ongoing; Obs, observational phase IV study; PMO, postmarketing obligation; RCT, randomized controlled trial; SAT, single‐arm trial. ^a^ Two obligations relating to the same RCT. ^b^ Four obligations (including 1 pooled analysis) relating to two SATs.

There was overall considerable heterogeneity between the EMA and the FDA with respect to the number of postmarketing studies required, their objectives, and design characteristics. Key characteristics of clinical postmarketing obligations at the aggregate level are presented in Table 4 (see the Appendix for additional details on postmarketing obligations). A comparison of postmarketing obligations at the level of drug‐indication pair is shown in Figures 2, 3, 4.

**Table 4:** Key Characteristics of Clinical Postmarketing Obligations (PMOs) Imposed by the EMA and the FDA

	EMA			FDA		
Criteria	CMA	Regular Approval	Overall	AA	Regular Approval	Overall
Number of Indications With Clinical PMOs	10	2	12	15	2	17
*Postmarketing obligation study design*						
Included at least one RCT	5	1	6 (50%)	13	1	14 (82%)
Included at least one single‐arm trial	4	1	5 (42%)	3	0	3 (17%)
Included at least one observational phase IV	5	1	6 (50%)	1	1	2 (12%)
*Postmarketing obligation study endpoint*						
Included at least one study with overall survival as primary endpoint	2	1	3 (25%)	3	1	4 (24%)
Included at least one RCT with overall survival as primary endpoint	0	1	1 (8%)	2	1	3 (18%)
*Postmarketing obligation status (December 2018)*						
All obligations fulfilled on time	1	0	1 (8%)	5	0	5 (29%)
All obligations fulfilled, with delays	4	1	5 (42%)	7	1	8 (47%)
Has open obligations, running on time	0	0	0 (0%)	1	1	2 (12%)
Has open obligations, running with delays	5	1	6 (50%)	2	0	2 (12%)

Abbreviations: AA, Accelerated Approval; CMA, Conditional Marketing Authorization; EMA, European Medicines Agency; FDA, US Food and Drug Administration; RCT, randomized controlled trial.

Overall, the EMA imposed clinical postmarketing obligations (i.e., obligations for confirmatory studies of clinical efficacy and/or safety in patients) for 12 of 17 approved indications (71%; 10 of these were CMA) and the FDA for 17 of 20 approved indications (85%; 15 of these were AA). The mean number of clinical postmarketing obligations for EMA CMAs was 2.3 (range, 1 to 4) and 0.6 (range, 0 to 3) for EMA regular approvals. It was 1.8 (range, 1 to 4) for indications approved through the FDA AA pathway and 0.4 (range, 0 to 1) for FDA regular approvals. There was only small overlap between the EMA and the FDA in individual studies used to address postmarketing obligations. Postmarketing obligations were addressed by 43 unique clinical studies, but only two of these were used for both EMA and FDA obligations.

Key characteristics of clinical postmarketing obligations are shown in Table 4. Postmarketing obligations included at least one RCT in six of the 12 EMA approvals with obligations (50%), compared to 14 of the 17 FDA approvals with obligations (82%). EMA approvals included single‐arm or observational studies for nine of 12 approvals (75%), whereas only five of 17 (29%) approved FDA indications with clinical confirmatory studies included these noncomparative study designs. Overall survival was a primary endpoint in postmarketing studies for only three of 12 approved EMA indications (25%) and four of 17 (24%) FDA indications. These studies were RCTs in one EMA and three FDA approvals.

Eighty‐seven percent of EMA postmarketing obligations after CMA were conducted in populations similar to the approved indication—that is, patients falling under the approved label or follow‐up studies of the pivotal trial. This was the case for only 59% of FDA obligations attached to AA, with the remaining 41% conducted in different populations, such as previously untreated patients or patients with different cancer types (data shown in the Appendix).

### Status of Postmarketing Obligations

We assessed the status of postmarketing obligations as of December 2018, resulting in a median follow‐up time of 7.25 years overall (median 6.6 years for EMA and 7.5 years for FDA approvals). After this time, half of EMA approvals with clinical postmarketing obligations had all requirements fulfilled. However, there were delays for five of these, and all obligations had been fulfilled on time for only one approved indication. The remaining 50% of approved EMA indications had open obligations, which were all running behind schedule. Of the 17 approved FDA indications with clinical postmarketing obligations, five (29%) had fulfilled all obligations on time, and obligations had been fulfilled with a delay for eight indications (47%). Two approved FDA indications had open obligations running on time, and two with delays. EMA obligations had a median delay of 10 months (range, no delay to five years), and FDA obligations had a median delay of zero months (range, no delay to three years), although this included five cases in which the manufacturer was released from an obligation and it was therefore considered fulfilled on time by the FDA. There were shorter delays for obligations for indications approved under EMA CMA or FDA AA (EMA: median six months; FDA: median zero months) compared to regular approvals (EMA: median 29 months; FDA: median 17.5 months).

### Postmarketing Evidence Generation

Among drug‐indication pairs that received approvals through both EMA CMA and FDA AA pathways (*n =* 4), EMA obligations included a total of nine nonrandomized studies (comprising follow‐up of pivotal single‐arm trials, new single‐arm trials, and observational safety studies) and a total of three RCTs, with no RCT imposed for two of the indications (Figure 2). In contrast, FDA obligations included one or two RCTs for each indication. The FDA considered the evidence generation obligations under AA for brentuximab vedotin fulfilled on the basis of the results from one of the required RCTs, while the drug remained under CMA provisions in Europe, with confirmatory studies ongoing and delayed by two to five years. For crizotinib, the same RCT was used in both Europe and the United States to confirm clinical benefit. Ofatumumab was also converted to regular approval by both agencies, although on the basis of different studies: the confirmatory study requested by the EMA was a new RCT conducted in patients with refractory disease (approved indication),^33^ whereas the confirmatory FDA RCT was conducted in the first‐line setting.^34^

Among drug‐indication pairs approved through regular EMA and FDA AA pathways (*n =* 7), postmarketing studies were imposed by the EMA for only two indications (lapatinib and pomalidomide), while the FDA imposed obligations for all indications (Figure 3). FDA obligations included a minimum of one RCT per indication, with the exception of ponatinib for acute lymphoblastic leukemia. By December 2018, the FDA considered postmarketing obligations fulfilled for all except one drug (lapatinib), with confirmatory evidence submitted to the FDA on time in two cases (dasatinib and nilotinib) and with delays ranging between 11 and 20 months for the remaining indications.

Among indications with EMA CMA and regular FDA approval (*n =* 5), the EMA imposed postmarketing studies for all conditionally approved indications, while the FDA imposed obligations for only two of them (vandetanib and vismodegib) (Figure 4). EMA obligations included an RCT for only two of five conditionally approved indications. Confirmatory studies for the remaining conditionally approved indications included observational phase IV studies, a follow‐up of the pivotal single‐arm trial, and a new single‐arm trial. Two of the drugs were converted to regular approval after delayed submission of confirmatory study reports (pazopanib and vismodegib), while two others remained under CMA after slow recruitment for confirmatory studies (bosutinib and vandetanib).^7^, ^35^

## Discussion

We reviewed regulatory outcomes, pathways, pivotal trial evidence, final approved indications, and postmarketing obligations of 21 matched cancer drug‐indication pairs for which early evidence is less complete than normally required at the time of approval, to assess differences in regulatory decisions under uncertainty between the EMA and the FDA. We found that both agencies showed an overall high acceptance of uncertainty, with the vast majority of applications in our sample of drugs with limited evidence at the time of market entry being approved by both regulators. Although both agencies overwhelmingly relied on the same evidence base, there were frequent discrepancies in the use of special (EMA CMA and FDA AA) vs. regular approval pathways across the two settings. Finally, we found marked differences in the design and objectives of postmarketing obligations imposed by the EMA and the FDA.

### Discrepancies in the Use of Special Regulatory Pathways

While both the EMA and the FDA were more likely to grant, rather than withhold, marketing approval for the cancer drug applications in our cohort of matched drug‐indication pairs, there was little concordance in the use of regulatory pathways. There were only four of 21 cases in which both the EMA and the FDA granted approval through CMA and AA pathways, respectively, for the same drug‐indication pair, while in 12 cases one agency granted approval through one of these special regulatory pathways, but the other granted regular approval.

The distinction between regular approval and approval through EMA CMA or FDA AA pathways is not trivial. For most of the 12 drug‐indication pairs in our sample with regular approval by one agency, additional evidence on efficacy and safety became available only through postmarketing obligations imposed by the other agency as a condition to granting approval (there were only four cases in which such evidence was requested by the agency granting regular approval). The case of ponatinib is helpful to illustrate the role of mandating the collection of postmarketing evidence. While the EMA granted regular approval on the basis of a single‐arm trial, the FDA granted approval through the AA pathway, in part due to a potential safety issue detected in that same single‐arm trial. The FDA therefore requested additional safety data from a postmarketing RCT, which had to be stopped early because of a higher rate of adverse events in the ponatinib treatment arm.^36^ As a consequence, the drug was temporarily withdrawn from the market in the United States and subsequently reintroduced with a revised, restricted indication.^37^ Prompted by the FDA action, the EMA also reviewed the additional evidence and concluded that the benefit‐risk balance remained positive.^38^ Nevertheless, the EMA review of whether additional evidence changed the benefit‐risk assessment was only possible because additional evidence had been collected under the FDA AA provisions.

This pattern of relevant evidence not materializing without regulatory obligation is not restricted to our sample of drugs: in a study of all FDA‐approved drugs between 2009 and 2012 that did not have FDA‐imposed postmarketing obligations, the majority of trials conducted after approval were found to be conducted in new indications (61%) or expanded populations of the approved indication (20%).^39^ Taken together, the findings of that study and ours suggest that, unless regulators impose the conduct of additional studies (typically through CMA at the EMA or AA at the FDA), the evidence on efficacy and safety in the intended population that is available at the time of market approval is all we will get. It is therefore important for policymakers to consider the potential consequences of a shift in evidence standards for initial approval. The routine granting of full approval for drugs with limited data on benefits and risks can create a precedent for a lower evidence threshold for drug approval being applied indiscriminately, with relevant additional evidence unlikely to materialize once the drug is on the market.

Our finding that the EMA and the FDA do not use CMA and AA approval congruently confirms and extends what other researchers have reported.^9^, ^21^, ^31^ We investigated possible reasons for these discrepancies. One potential reason relates to the fact that the EMA can grant CMA only for first (or initial) marketing authorizations, but not for any subsequent changes, such as extensions or variations of existing marketing authorizations. In these situations, the EMA faces a decision to grant approval (if the benefit‐risk balance is deemed positive) or not (if it is negative). The evidence in our sample points toward a more lenient approach by the EMA in these cases: approval was granted in four cases, whereas no approval was given in only one of the cases in which the statutory limitation on CMA applied.

Another possible explanation for the observed discrepancy in granting approval through CMA/AA vs. regular pathways is that the EMA and the FDA based their assessments on different evidence. A study of concordance between the EMA and the FDA across all therapeutic areas from 2014 to 2016 found overall very good agreement between the two agencies for approval vs. no approval decisions; but, similar to our study findings, there was considerable disagreement in the use of special approval pathways.^31^ The study found that discordance in the use of regulatory approval pathways was in equal parts due to differences in the conclusions on efficacy drawn from the same clinical data submitted to both regulators and to additional clinical data reviewed by one agency but not the other. The situation was somewhat different in our study. In our sample of 21 cancer drug‐indication pairs with limited evidence on efficacy and safety at the time of market entry, there were only two cases in which different pivotal trials were submitted to the EMA and the FDA—all others relied on the same clinical trial evidence but resulted in approval through different pathways and discrepant regulatory outcomes.

In a qualitative study with representatives from the EMA and the FDA, respondents attributed divergent opinions on cancer drugs to the fact that the EMA regarded PFS as clinical benefit in itself, whereas the FDA saw it as a surrogate endpoint that would need to be confirmed by additional studies.^32^ Such discrepant views could explain a situation where the same evidence package leads to the FDA utilizing the AA pathway and the EMA granting regular approval. However, we found only one case (lapatinib) in which different views on PFS could have explained approval through regular EMA and FDA AA pathways. Coincidentally, this was a variation of an existing marketing authorization, and the discrepancy in regulatory pathways may also have been due to statutory limitations for EMA's use of CMA.

### Evidence Standards for Regular Approval

An important finding of our study is that, despite discrepancies in the use of EMA CMA and FDA AA, there was an overall high acceptance of uncertainty for the approval of cancer drugs by both the EMA and the FDA. Our sample consisted exclusively of drug‐indication pairs for which at least one of the two regulatory agencies considered the evidence base (composed of the same pivotal trials in 19 cases) insufficient to grant regular, or full, approval. Nevertheless, for more than half of the 21 matched drug‐indication pairs, one of the agencies did grant regular approval, indicating that regulators often did not consider it necessary to impose obligations to collect additional evidence under the framework of CMA or AA. In addition, regulators were far more likely to grant approval than to deny it: there were only five drug‐indication pairs for which one of the agencies did not grant approval (four of the negative decisions coming from the EMA and only one from the FDA). In many cases, the exception of approving a drug with limited clinical evidence on safety and efficacy has therefore become the norm. Our findings complement and extend research by others about exceptionalism in drug approval.^40^, ^41^ Policymakers need to be aware of de facto (as opposed to statutory) evidence standards applied by regulatory agencies to enter an informed discussion about whether this is a desired trajectory for regulating the medicinal products market. Granting regular approval on the basis of early efficacy and safety data can affect the regulatory landscape overall by signaling to pharmaceutical companies that evidence that was historically considered for approval through special regulatory pathways (EMA CMA or FDA AA) may be sufficient for regular approval.

Our findings further suggest that postmarketing obligations imposed by regulators were unlikely to resolve unanswered questions concerning efficacy and safety for a number of reasons.

### Issues With Timely Evidence Generation in the Postmarketing Setting

First, there were often delays, sometimes substantial, in the conduct of studies and submission of results from postmarketing obligations. Sixty‐one percent of EMA postmarketing obligations and 40% of FDA obligations in the CMA and AA pathways, respectively, were submitted with a delay or were ongoing behind schedule. This is broadly in line with previous studies of EMA postmarketing obligations.^42^, ^43^ For the FDA, our finding of a rate of 7% delayed ongoing studies is only half of that found in a 25‐year investigation of AA,^25^ but this may be explained by the difference in time frames as well as play of chance, given that we identified only three ongoing studies for drugs approved under the FDA AA pathway. Overall, our study adds to previously voiced concerns about the timeliness of postmarketing studies conducted as part of special approval pathways.^14^, ^16^

Policymakers should aim to create a regulatory environment in which pharmaceutical companies are incentivized to produce this evidence. Currently, there appears to be little to lose for companies once their product has been approved. While some legal instruments exist in both the EU and the United States to enforce compliance with postmarketing obligations (see Box), these are rarely used. The threat of revoking approval remains a tame one in the United States, where FDA officials appear unwilling to withdraw market approval due to lack of proven efficacy in a lengthy and resource‐intensive process that invokes an image of a regulator blocking patients from accessing an effective treatment.^2^ Stricter enforcement of compliance with postmarketing obligations could improve the rate of timely fulfilled obligations and lead to complete evidence packages becoming available for drugs with EMA CMA or FDA AA. Regulators need to have the mandate to ensure compliance with postmarketing obligations as part of their mission to protect public health and ensure the availability of high‐quality, safe, and effective medicines for citizens.^44^, ^45^ In addition, companies could be incentivized to ensure timely completion of postmarketing studies by making up‐to‐date status reports on postmarketing obligations and their results publicly and easily available. Although such a database has been introduced in the United States, it does not appear to be up‐to‐date and complete. An equivalent database for the EU is missing.

### Issues With Robust Study Designs in the Postmarketing Setting

The second reason postmarketing obligations were unlikely to provide adequate answers to open questions about efficacy and safety is that regulators appear to face a trade‐off between receiving confirmatory evidence either from robustly designed studies (i.e., RCTs) or studies conducted in populations matching the approved indication, but not both. We found that the EMA routinely accepted noncomparative studies to generate confirmatory data, even in cases where CMA was granted on the basis of single‐arm trials (bosutinib, brentuximab vedotin, and vismodegib), while FDA postmarketing obligations more typically consisted of RCTs. The lack of a control in noncomparative studies hinders causal interpretation of observed treatment effects. The suitability of such study designs to establish or confirm a positive benefit‐risk ratio of conditionally approved drugs is therefore questionable, as is reflected in conventional grading of evidence frameworks and the default study design preferences of regulatory agencies.^20^, ^46^, ^47^, ^48^, ^49^ At the same time, the vast majority (87%) of EMA postmarketing studies were planned to be conducted in the same patient population for which approval was granted, while the FDA accepted confirmatory studies conducted in a different setting for a substantial minority (41%) of cases. This highlights an important difference in the two agencies’ approach to postmarketing evidence generation: while the EMA has a demonstrated preference for confirmatory evidence to come from the same population as the approved indication, the FDA allows confirmatory studies to be conducted in a different population (typically in patients with less advanced disease) in an effort to ensure postmarketing obligations are fulfilled.^20^ The FDA's policy reflects considerations that RCTs may be difficult or unethical to conduct in rare diseases or populations with no available therapy (although these arguments can be challenged by examples of RCTs being conducted in rare diseases and under challenging conditions^50^, ^51^). Importantly, approval itself can be turned into an argument against conducting an RCT to confirm the clinical benefit of a new drug. Regulatory approval signals a positive benefit‐risk balance to patients and physicians, even if based on preliminary data, and creates ethical challenges for withholding the drug from patients when conducting a confirmatory trial in the approved indication in a setting where the drug has become available. However, failure to conduct a robust study in the intended patient populations results in the availability of a drug on the market without confirmed positive benefit‐risk ratio; in other words, a different evidence standard is being applied where preliminary trial results are deemed sufficient. In these circumstances, it is important to consider the uncertainty surrounding the evidence base for initial (special) approval. Special, rather than regular, approval pathways are used when there is a need for confirmation of an indicative positive benefit‐risk ratio. The strongest confirmatory evidence will come from robust studies demonstrating a causal positive treatment effect in the population that the drug is intended for.

To address issues with postmarketing study designs, it is instructive to revisit the original AA model, introduced in the United States in 1992 to address the evidence vs. access conundrum when promising treatments emerged to treat HIV/AIDS patients. The FDA has described the “ideal approach” to AA as following up pivotal, randomized trials in the postmarketing phase to obtain confirmatory evidence using patient‐relevant outcomes after initial approval based on a surrogate measure.^20^ However, this standard was abandoned by the FDA due to concerns about the feasibility of continuing an RCT while the experimental drug was already available.

Although we found that the EMA and the FDA routinely deviated from the gold standard for confirmatory studies by either accepting weak study designs (EMA) or different populations (FDA), our sample also included a case that illustrates that the original model of obtaining confirmatory evidence from an RCT in the intended population was still possible, if only requested by the regulatory agency. Ofatumumab was approved through both the EMA CMA and the FDA AA pathways for treatment of refractory chronic lymphocytic leukemia on the basis of a single‐arm trial. Both agencies imposed a confirmatory RCT as a postmarketing obligation. However, while the FDA accepted a confirmatory RCT in an earlier treatment setting, the confirmatory RCT requested by the EMA was in the same population as the approved indication. This study was completed in a timely manner. Upon its completion, the EMA converted from CMA to regular approval. (Coincidentally, the RCT had failed to meet its primary endpoint of improved PFS by independent review committee and also failed to demonstrate improved overall survival with ofatumumab compared to physician's choice of therapy, although it showed improved PFS by investigators’ assessment and time to next therapy.^33^)

### Issues With Study Endpoints in the Postmarketing Setting

Another reason postmarketing studies in our sample may not adequately address uncertainty around the benefit‐risk ratio is the use of surrogate measures, such as response rate or PFS, instead of patient‐relevant outcomes, such as overall survival or quality of life. Although surrogate measures are potentially a useful tool to shorten clinical trial duration if they reliably predict clinical benefit, the validity of even widely used surrogates such as PFS in cancer often remains unproven.^52^, ^53^ When surrogate measures are used for regulatory approval of cancer drugs, they are typically nonvalidated in both Europe and the United States.^54^, ^55^ In our sample, the most commonly used primary endpoint in clinical postmarketing studies of drugs approved through the EMA CMA or FDA AA pathway was PFS, followed by response rate. Our results show that, for drugs with less complete efficacy data than usually required at the time of approval, it is unlikely that robust overall survival data become available in the postmarketing setting. Postmarketing obligations included overall survival as primary endpoint for only 25% and 24% of EMA and FDA approvals, respectively. Moreover, all EMA obligations after CMA with overall survival primary endpoints were single‐arm studies, which are not suitable for measuring time‐to‐event endpoints due to the lack of a comparator. Our study adds to other research showing that clinical studies in the postmarketing setting are likely to measure surrogate outcomes,^14^ including the same measures that were used for initial approval in special regulatory pathways.^7^, ^25^

There is now a body of literature, including this study, demonstrating that postmarketing studies are often delayed in both the EU and the United States,^14^, ^16^, ^42^ do not commonly use robust study designs or patient‐relevant endpoints,^14^, ^24^, ^25^, ^56^ and are subject to substantial deviations from initially imposed requirements.^43^, ^57^ We therefore echo concerns previously voiced by others about placing too much emphasis on postmarketing studies to address uncertainties in the evidence submitted at the time of market approval.^42^, ^58^ Given the importance of postmarketing evidence generation as an integral component of EMA CMA and FDA AA, shortcomings in the design and conduct of studies in this setting raise the question whether these special approval pathways work as intended.

### Limitations

Our study covers cancer drugs with EMA outcomes from 2009 to 2013 inclusive. We chose the time frame in order to allow for sufficient time to complete postmarketing obligations and take the current status of EMA CMAs and FDA AAs into consideration. The time period of our study allowed for a minimum of five years to complete and submit postmarketing studies, in line with previous research.^56^

Our study was limited to cancer drugs. This is by far the largest group of therapeutic agents receiving EMA CMA and FDA AA.^7^, ^13^ Despite the focus on one therapeutic area, our findings are therefore highly relevant for other products that benefit from these special approval pathways. A recent example is hydroxyprogesterone caproate, a drug for preventing preterm births, which had received FDA AA but failed to demonstrate effectiveness in a confirmatory trial, leading consumer rights advocates to call for its withdrawal from the market.^59^

The focus on cancer drugs with regulatory outcomes from 2009 to 2013 led to a sample of 21 drug‐indication pairs. We considered the limitations of a small sample size to be more than offset by the opportunity to investigate the evidence requirements and regulatory decisions for each in more detail. Focusing on a five‐year period allowed us to investigate regulatory decision making under uncertainty, including both preapproval and postapproval evidence requirements. Furthermore, we included all cancer drug‐indication pairs in the chosen time period for which either EMA CMA or FDA AA was granted. Our sample therefore represents a full overview of regulatory decisions for cancer drugs for which at least one regulator considered the evidence on efficacy and safety insufficient for regular approval. Due to negative FDA decisions not being published systematically, we did not include cases where the FDA denied approval and an application for EMA approval was never submitted. However, this is in line with the aims of this study, since we were only interested in drug‐indication pairs for which a regulatory outcome by both the EMA and the FDA existed.

Our sample included variations of existing approvals, for which the EMA cannot grant approval through the CMA pathway. Although this represents an important difference in how the two regulatory bodies can manage uncertainty, our study fully took this statutory restriction into account by explicitly considering a range of regulatory tools, including outcomes (approval vs. no approval decision), pathways (regular approval vs. approval through a special pathway), final approved indications, and postmarketing obligations, instead of focusing on the regulatory pathway alone.

There were also limitations in the information we were able to extract. We relied on publicly available documents, but found that these sometimes lacked important details. For example, for most approvals for variations of existing drugs, the FDA does not provide review documents. We also had difficulties ascertaining the status of some postmarketing obligations. With no comprehensive, up‐to‐date database of postmarketing obligations available, information on the status of these studies had to be traced through a variety of sources, as described in the methods section. We could determine only whether obligations were considered fulfilled by the regulatory agency—this included instances where the manufacturer was released of an obligation, and the study was never completed, and at least one case where a trial demonstrated inferiority of the conditionally approved drug,^33^ yet the obligation to conduct the study was considered fulfilled.

### Conclusions

US and European drug regulators were often willing to grant regular approval to cancer drugs for which data on efficacy and safety were less complete than usually required, rather than deny approval or require the collection of additional evidence through AA and CMA pathways, respectively. When postmarketing studies were imposed, shortcomings in their design and delayed submission of results raise questions over the ability of the FDA's AA and the EMA's CMA to reconcile early market access with maintaining rigorous regulatory standards.

*Funding/Support*: Work on this study was supported in part by funding from Health Action International. The funder had no role in the design and execution of this study, interpretation of its results, and decision to submit this work to be published.

*Conflict of Interest* Disclosure: All authors completed the ICMJE Form for Disclosure of Potential Conflicts of Interest. Huseyin Naci reported support from the Commonwealth Fund outside the published work. No other conflicts reported.

## Supporting information


Online Appendix
