# Supplementary material for: Approval of Cancer Drugs With Uncertain Therapeutic Value: A Comparison of Regulatory Decisions in Europe and the United States
Source: Milbank Q. 2020 Oct 6;98(4):1219–56. doi: 10.1111/1468-0009.12476 (PMC7772660; doi:10.1111/1468-0009.12476)
Supplement: Supplementary file 1 — Online Appendix [file MILQ-98-1219-s001.pdf]

APPENDIX

Approval of cancer drugs with uncertain therapeutic value: a comparison of regulatory decisions in Europe and the United States

Maximilian Salcher-Konrad, MSc *[1,2,3] Huseyin Naci, PhD [1] Courtney Davis, PhD [4]

[1] Department of Health Policy, London School of Economics and Political Science, London, United Kingdom.
[2] Care Policy and Evaluation Centre (CPEC), London School of Economics and Political Science, London, United Kingdom.
[3] LSE Health, London School of Economics and Political Science, London, United Kingdom.
[4] Department of Global Health & Social Medicine, King's College London, London, United Kingdom.

1. Compilation of information on post-marketing obligations and their current status

For EMA approvals, information on imposed post-marketing studies and their status was extracted from the EPAR, a previous report compiled by the EMA on CMA,[1] and three trial registries (clinicaltrials.gov; EU Clinical Trials Register; and EU PAS Register). We also reviewed publicly available meeting minutes of two EMA committees responsible for monitoring post-marketing obligations (PRAC and CHMP committees) to clarify information provided in other documents. Characteristics of post-marketing studies were extracted primarily from trial register entries, and from the EMA 10-year report on CMA. If no information was available from either of these sources, we relied on information included in the EPAR. We only extracted imposed post-marketing studies listed in the Annex II to the marketing authorisation under "obligations" (or "specific obligations" in the case of CMAs). The status of the obligations was determined primarily through the EMA 10-year report on CMA. For obligations that had not been fulfilled at the time of the cut-off date for the EMA report, we first reviewed the "steps taken after marketing authorisation" document on the EMA's website for references to obligations being fulfilled. We also accessed historical versions of the Annex II for each product to review changes to the list of obligations to determine whether individual obligations were considered fulfilled and therefore removed from the Annex II. For dates of fulfilment of obligations, we primarily relied on the dates of submission of study reports given in the EMA 10-year report on CMA. For obligations that had not been fulfilled at the cut-off date for this report, we searched the "steps taken after marketing authorisation" document and publicly available meeting minutes of the PRAC and CHMP committees for dates when reports on obligations were submitted. When a study report was discussed in one of the meeting minutes, we assumed that submission of the report was made after the prior meeting of that committee (committees meet monthly). If a submission date could not be identified through these sources, we took the actual primary completion date or actual completion date (depending on the endpoint of interest of the obligation) of clinical trials register entries as proxy for the submission date. Finally, if no clinical trials register entry existed or was not updated, we used the midpoint between the effective date of the last Annex II version that contained the obligation and the effective date of the first Annex II version where it had been removed as a proxy for the submission date.

For FDA approvals, information on post-marketing study requirements (i.e. studies subject to reporting requirements) and their status was extracted from the initial approval letter, label changes, the FDA's online database on post-marketing commitments and requirements, and clinicaltrials.gov. Characteristics of post-marketing studies were extracted primarily from clinicaltrials.gov. If no clinicaltrials.gov entry could be identified, we relied on the FDA's database. As a minimum, we extracted information on required characteristics from approval letters and review documents. The status of post-marketing studies was determined primarily by perusing all label changes and associated approval letters for the drug in question. We also queried the FDA's database of post-marketing commitments and requirements. Where the status of the requirements could not be determined through approval letters, label changes and the FDA database, we relied on the study completion status on clinicaltrials.gov. Finally, we obtained the date of fulfilment of the requirement. We primarily relied on the submission dates for study reports submitted to the FDA, as indicated in supplemental approval letters. We also checked the FDA's database of post-marketing commitments and requirements for completion dates. In cases where submission dates of study reports could not be determined from these sources, we used the actual primary

completion date or actual completion date (depending on the endpoint of interest for the requirement) as stated on clinicaltrials.gov as a proxy for timeliness of study report submission to the FDA.

2. Characteristics of clinical post-marketing study obligations

	EMA		FDA	
	**CMA**<br>**N (%)**	**Regular approval**<br>**N (%)**	**AA**<br>**N (%)**	**Regular approval**<br>**N (%)**
**Total number of obligations**	**23**	**4**	**27**	**2**
Required under CMA/AA	22 (96%)	0 (0%)	21 (78%)	0 (0%)
Other obligations	1 (4%)	4 (100%)	6 (22%)	2 (100%)
Focus of post-marketing study				
Focus on efficacy and safety	10 (43%)	1 (25%)	7 (26%)	0 (0%)
Focus on efficacy only	10 (43%)	2 (50%)	15 (56%)	0 (0%)
Focus on safety only	3 (13%)	1 (25%)	5 (19%)	2 (100%)
Post-marketing study design				
RCT	8 (35%)	2 (50%)	22 (81%)	1 (50%)
Single-arm trial	9 (39%)	1 (25%)	4 (15%)	0 (0%)
Observational Phase IV	6 (26%)	1 (25%)	1 (4%)	1 (50%)
Active control arm	8 (35%)	1 (25%)	13 (48%)	0 (0%)
Placebo control arm	0 (0%)	1 (25%)	8 (30%)	1 (50%)
Double blind	0 (0%)	1 (25%)	4 (15%)	1 (50%)
Open label	22 (96%)	3 (75%)	21 (78%)	1 (50%)
Post-marketing study endpoint				
Overall survival	3 (13%)	1 (25%)	4 (15%)	1 (50%)
Progression-free survival	11 (48%)	1 (25%)	11 (41%)	0 (0%)
Adverse events	6 (26%)	2 (50%)	3 (11%)	0 (0%)
Response rate	7 (30%)	1 (25%)	5 (19%)	0 (0%)
Other surrogate endpoint	0 (0%)	1 (25%)	4 (15%)	0 (0%)
Unclear endpoint	0 (0%)	0 (0%)	1 (4%)	1 (50%)
Post-marketing study population				
Study population similar to approved indication	20 (87%)	2 (50%)	16 (59%)	1 (50%)
Study population different to approved indication	3 (13%)	2 (50%)	11 (41%)	1 (50%)
Status (December 2018)				
Ongoing, on track	0 (0%)	0 (0%)	1 (4%)	1 (50%)
Ongoing, delayed	6 (26%)	1 (25%)	2 (7%)	0 (0%)
Fulfilled, on time	9 (39%)	1 (25%)	13 (48%)	0 (0%)
Fulfilled, delayed by ≤1 year	6 (26%)	0 (0%)	6 (22%)	0 (17%)
Fulfilled, delayed by ≤2 years	0 (0%)	1 (25%)	3 (11%)	0 (0%)
Fulfilled, delayed by ≤3 years	0 (0%)	1 (25%)	0 (0%)	1 (50%)
Fulfilled, delayed by ≤4 years	2 (9%)	0 (0%)	0 (0%)	0 (0%)
Status unclear	0 (0%)	0 (0%)	2 (7%)	0 (0%)

Reference

1. European Medicines Agency. *Conditional Marketing Authorisation: Report on Ten Years of Experience at the European Medicines Agency*. London; 2017. http://www.ema.europa.eu/docs/en_GB/document_library/Report/2017/01/WC500219991.pdf.
